# Efficacy and short-term outcomes of preoperative chemoradiotherapy with intermittent oral tegafur-uracil plus leucovorin in Japanese rectal cancer patients: a single center experience retrospective analysis

**DOI:** 10.1186/s12957-017-1177-5

**Published:** 2017-05-31

**Authors:** Ryosuke Nakagawa, Yuji Inoue, Takeshi Ohki, Yuka Kaneko, Fumi Maeda, Masakazu Yamamoto

**Affiliations:** Department of Surgery, Institute of Gastroenterology, Tokyo Women’s Medical University, 8-1 Kawada-cho, Shinjuku-ku, Tokyo, 162-8111 Japan

**Keywords:** UFT/LV, Intermittent, Rectal cancer, Preoperative chemoradiotherapy, Oncologic outcomes

## Abstract

**Background:**

Various types of preoperative chemoradiotherapy (CRT) have been established for rectal cancer; thus, Physicians will need to refine the selection of appropriate preoperative CRT for different patients since there are various treatment regimens. Oral tegafur-uracil (UFT) plus leucovorin (LV) is commonly used to treat rectal cancer in Japan. Oral chemotherapy offers patients many potential advantages. Since 2008, we have been performing preoperative CRT with intermittent oral UFT plus LV in locally advanced rectal cancer patients to prevent postoperative local recurrence. Here, in a retrospective analysis, we evaluated the efficacy and short-term outcomes of preoperative CRT with intermittent oral UFT plus LV.

**Methods:**

We analyzed data from 62 patients with locally advanced rectal cancer, including 31 patients who underwent preoperative CRT between 2009 and 2013 (the CRT group) and 31 patients who were treated with surgery alone between 2001 and 2008 (the non-CRT group). Clinicopathologically, both groups included patients with rectal cancer at clinical tumor stages III-IV or clinical node stages 0-III. In the CRT group, curative operations were performed ≥8 weeks after CRT. Patients were concomitantly treated with 2 cycles of oral UFT (300 mg/m^2^/day, days 1–14 and 29–42) plus LV (75 mg/day, days 1–14 and 29–42) and 45 Gy of radiotherapy. Chemotherapy was repeated every 28 days, followed by a 2-week break.

**Results:**

The completion rate of CRT was high at 94% (*n* = 29/31). The downstaging rate of CRT was 61% (*n* = 19/31). The pathological complete response rate was 6.5% (*n* = 2/31). Significant differences were observed in the 3-year local recurrence rate between the two groups (*P* < 0.05).

**Conclusions:**

Preoperative CRT with intermittent oral UFT plus LV appears to be a tolerable and effective treatment for Japanese patients with rectal cancer. A further investigation of a diversification of preoperative CRT for Japanese rectal cancer patients is required.

## Background

Oral chemotherapy offers patients many potential advantages. For example, a patient who is receiving oral chemotherapy may be able to return to work faster than one who is receiving an intravenous cancer treatment. Burdening the patient with central venous catheter placement and infusion pumps is also avoided with the use of oral chemotherapy. Self-administration also means fewer trips to the hospital or the doctor’s office. Moreover, patients receiving intravenous cancer treatments may develop complications with the infusion, clotting, or infections [1]. Studies have shown that most patients prefer oral chemotherapy to intravenous therapy, as long as efficacy is not compromised [2, 3]. Moreover, from 2000 to 2050, the number and percentage of Japanese more than 70 years old are expected to double. This increase will be accompanied by a marked increase in patients with cancer, requiring elderly care. The cancer incidence exponentially increases with advancing age, therefore the number of older patients with rectal cancer will surge. Managing rectal cancer treatment in the elderly is an increasing problem. There are several reasons that make it more difficult for elderly patients to withstand chemotherapy, including decreased renal, hepatic, respiratory, and cardiac functions; a decreased bone marrow reserve; a different distribution and clearance of drugs; and an increased probability of comorbidities [4].

Tegafur-uracil (UFT) is a prodrug of 5-fluorouracil (5-FU). The oral administration of tegafur and uracil combined in a 1:4 molar ratio simulates the continuous intravenous administration of 5-FU [5, 6]. Oral UFT plus leucovorin (LV) is commonly used to treat rectal cancer in Japan. Ota et al. [7] reported that UFT administered at doses of 300–600 mg/day is extremely well tolerated and found evidence of anti-tumor activity in a various solid tumors. We assert that the oral UFT regimen can be considered a well-grounded therapeutic option in patients who are unable to withstand combination treatment (e.g., oxaliplatin) or in those in whom oral treatment is preferable for several reasons (e.g., psychological, clinical, or compliance problems). In Japan, total mesorectal excision (TME) plus lateral lymph node resection (LLND) has become the standard surgical treatment for Stage II and Stage III primary rectal cancer according to the Japanese Society for Cancer of the Colon and Rectum (JSCCR) guidelines [8]. However, in some Japanese institutes preoperative chemoradiotherapy (CRT) has been performed as part of a clinical trial. In our retrospective study of Japanese patients at a single center, we compared preoperative CRT using intermittent oral UFT plus LV to surgery alone, evaluating the efficacy, toxicity, and 3-year outcomes.

## Methods

### Study participants

**Fig. 1:**
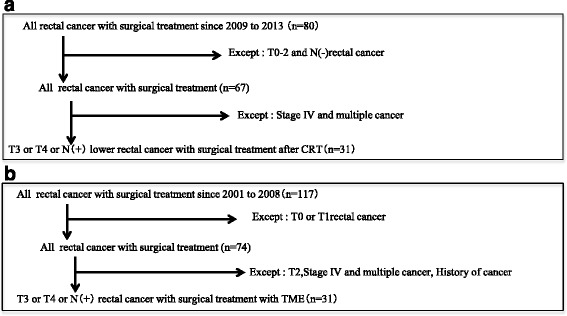
Flow chart of the study design. **a** Chemoradiotherapy (CRT) group. **b** Non-CRT group

Sixty-two patients with rectal cancer at clinical tumor stages (cT) 3–4 or clinical node stages (cN) 0–2 underwent curative operations between 2001 and 2013 at the Department of Surgery, Institute of Gastroenterology, Tokyo Women’s Medical University, Tokyo, Japan. We have been performing preoperative CRT at this institution since December 2008 in patients with rectal cancer at cT stages 3–4 or cN stages 0–2 to prevent postoperative local recurrence. The CRT group included 31 patients that between 2009 and 2013 were administered preoperative CRT with intermittent oral UFT plus LV. Surgery alone was performed between 2001 and 2008 in another 31 patients with rectal cancer at cT stages 3-4 or cN stages 0-2, which comprised the non-CRT (control) group (Fig. 1). We evaluated the efficacy, toxicity, downstaging (DS) rate, and 3-year outcomes associated with preoperative CRT with intermittent oral UFT plus LV. In addition, we compared the patient characteristics, postoperative complications, clinicopathological findings, and prognoses of the CRT and non-CRT groups. Every patient was classified according to the Union for International Cancer Control (UICC) 7^th^ edition TNM staging system.

All participants provided written informed consent before the study commenced. The study protocol was approved by the Institutional Review Board of the Tokyo Women’s Medical University, Tokyo, Japan (University Hospital Medical Information Network identifier UMIN000018563), and registered with Infrastructure for Academic Activities (https://upload.umin.ac.jp/cgi-open-bin/ctr_e/ctr_view.cgi?recptno=R000021481). Research was conducted in accordance with the principals of the 1964 Declaration of Helsinki and its later amendments.

### Imaging analysis

Computed tomography (CT) or magnetic resonance imaging (MRI) was used to evaluate tumor infiltration and the presence of lymph nodes (e.g., paraintestinal lymph nodes, lateral pelvic lymph nodes [LLNs]) >1 cm in diameter in the shortest dimension, or lymph nodes with clinical characteristics suggestive of metastasis. CT of the abdomen and radiography or CT of the thorax were performed to assess the extent of distant metastasis and dissemination.

### Chemotherapy

**Fig. 2:**
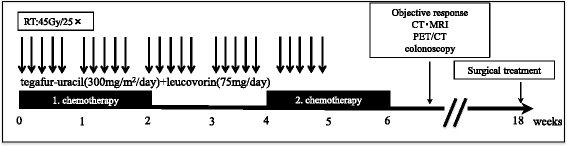
Treatment course of preoperative chemoradiotherapy (CRT). Preoperative chemotherapy consisted of two 2-week courses of tegafur-uracil (UFT; 300 mg/m^2^/day) and leucovorin (LV; 75 mg/ day) with a 2-week break. Radiation therapy was administered on weekdays for 5 consecutive weeks. Patients were monitored by interview, physical examinations, and blood tests every 2 weeks

An overview of the CRT protocol performed at our institution is illustrated in Fig. 2. The oral UFT plus LV regimen consisted of 2 cycles, every 3 weeks. The dose of UFT was 300 mg/m^2^/day, and the dose of LV was 75 mg/day on days 1–14. Chemotherapy was intermittently repeated every 28 days, followed by a 2-week break.

### Radiotherapy

Radiotherapy (RT) consisted of 45 Gy delivered in fractions of 1.8 Gy/day on weekdays for 5 consecutive weeks concomitantly with chemotherapy. RT was administered with a linear particle accelerator (Clinac 21EX; Varian Medical Systems, Inc., Palo Alto, California, USA), and radiation treatment planning was completed using the Eclipse™ system (version 7.3.10; Varian Medical Systems, Inc.).

A radiotherapist determined the treatment plan based on CT. Preoperative RT (total dose of 45 Gy) was delivered as photons with a 10-MV linear accelerator in 25 fractions (1.8 Gy/day; 5 fractions per week). RT was delivered to the pelvis through individually shaped portals, using a 3-field or 4-field box technique (anterior field, posterior field, and left and right lateral fields).

Patients were monitored through an interview, physical examinations, and blood tests every 2 weeks.

### Surgical procedures

At our institution, laparoscopic surgery for rectal cancer patients after preoperative CRT has been performed since 2012. Therefore, only open surgery was performed for rectal cancer patients after preoperative CRT during 2001 to 2011. Surgery was performed ≥8 weeks following the completion of preoperative CRT. Patients underwent curative operations for rectal cancer at the Department of Surgery, Institute of Gastroenterology, Tokyo Women’s Medical University, Tokyo, Japan. A case of non-curative resection was excluded from our study. Depending on the size of the tumor and its distance from the anal verge, the procedure was planned and performed by a surgeon. The indication to perform intersphincteric resection (ISR) was determined to maintain a 2-cm distal margin based on the preoperative assessment. The surgical procedure involved a LLND using a standard TME technique. At our institution, if the size of the LLN was >1 cm in diameter with the shortest diameter on preoperative CT or MRI, LLND was only performed for curative treatment. That is, LLND was only performed on the side with the enlarged LLN for curative treatment irrespective of whether the LLNs size decreased after CRT.

### Statistical analyses

The rates of overall survival (OS) (calculated from the date of surgery until death) and disease-free survival (DFS) (calculated from the date of surgery until rectal cancer recurrence) were determined using the Kaplan-Meier method. Comparisons between survival curves were performed using the log-rank test. Statistical analyses were conducted using JMP Pro 11 (SAS Institute Inc., Cary, NC, USA). *P*-values <0.05 were considered statistically significant.

## Results

A summary of the characteristics of the patients in the CRT and non-CRT groups is presented in Table 1 *.* The median follow-up period was 53.4 months (range, 18.2-79.2 months) in the CRT group. Curative operations were performed for all 31 patients in the CRT group. No significant differences were observed between the two patient groups with respect to clinical backgrounds, except for the type of operative procedure (*P <* 0.05). This may be explained by the introduction of laparoscopic surgery at our institute around 2005.

**Table 1:** Patient characteristics

	CRT(*n* = 31)	non-CRT(*n* = 31)	*P*-value
Gender(male: female)	24: 7	20: 11	0.263
Median age(range)	61(42-84)	61(30-92)	0.878
PS (0/1/2/3)[%]	(11[35]/18[58]//0[0]/2[7])	(10[32]/16[52]/1[3]/4[13])	0.608
ASA (1/2/3)[%]	(7[22]/21[68])/3[10])	(13[42]/15[48])/3[10])	0.246
cT(2/3/4)	2/25/4	0/26/5	0.344
cN(+/-)	24/7	21/10	0.393
Open: Laparoscopy	13: 18	31: 0	<0.05
Abdominoperineal resection(%)	17(55)	22(71)	0.188
Sphincter preserving operation(%)	14(45)	9(29)	0.188
LLND(%)	2(6.5)	1(3.2)	0.554
Completion rate of CRT(%)	29(94)	N/A	N/A

*PS* Performance status, *ASA* American Society of Anesthesiologist, *cT* clinical tumor stages, *cN* clinical node stages, *LLLD* Lateral lymph nodes dissection, *CRT* Chemoradiotherapy N/A not applicable

### Efficacy and downstaging

The median preoperative size of the tumors in the CRT group was 3.6 cm (range, 2.4–4.8 cm). The median postoperative size of the tumors was 2.2 cm (range, 1.1–4.1 cm). The median tumor volume reduction rate was 40% (range, 0.8–66%). The CRT response was objectively evaluated based on the Response Evaluation Criteria in Solid Tumors (RECIST) guidelines, version 1.1 [9]. A complete response was defined as a total disappearance of all lesions on a follow-up CT or MRI. Partial responses and progression of the disease were defined as a decrease of ≥30% or an increase of ≥20% in the total diameter of the target tumor lesions, respectively. The complete or partial response rate based on the RECIST criteria was high at 81% (*n* = 25/31). The correlations between the clinical stage and pathological stage are shown in Tables 2, 3 and 4. Nineteen of 31 (61%) patients in the CRT group showed DS (Table 2). DS of T-stage lesions was observed in 13 of 31 (42%) patients in the CRT group (Table 3), and DS of N-stage lesions was observed in 16 of 31 (52%) patients in the CRT group (Table 4).

**Table 2:** DS results in the patients with CRT (*n* = 31)

	pStage 0	pStage I	pStage II	pStage III	DS(%)
cStage 0	0	0	0	0	
cStage I	0	0	0	0	
cStage II	1	4	2	0	5/7(71)
cStage III	1	4	9	10	14/24(58)
total					19/31(61)

**Table 3:** DS results (T Stages)

	pT0	pT1	pT2	pT3	pT4	DS(%)
cT2	1	0	0	1	0	1/2(50)
cT3	1	0	7	16	1	8/25(32)
cT4	0	0	1	3	0	4/4(100)
total						13/31(42)

**Table 4:** DS results (N Stages)

	pN0	pN1	pN2	DS(%)
cN0	7	0	0	0/7
cN1	10	5	2	10/17(59)
cN2	3	3	1	6/7(86)
total				16/31(52)

### Toxicity grading

Drug toxicities observed in the CRT group (graded according to the Common Terminology Criteria for Adverse Events, version 4.0) are presented in Table 5. Three (9.6%) patients had grade 3 diarrhea. In two of these three patients, intermittent oral UFT plus LV administration needed to be discontinued, but RT was continued. During CRT, the most frequently reported toxicity was diarrhea, affecting 18 of 31 (58%) patients in the CRT group. No instances of leucopenia were observed, and there were no grade 4 events. The remaining 29 patients completed the CRT regimen. Thus, the CRT completion rate was high, i.e., 29 of 31 (94%) patients, as shown in Table 1.

**Table 5:** Toxicity Grade^a^ patients with CRT (*n* = 31)

	Grade 1	Grade 2	Grade 3	Grade 4	total(%)
Diarrhea	6	9	3	0	18(62)
Radiation dermatitis	4	2	0	0	6(21)
Nausea	1	0	0	0	1(3.4)
Leucopenia	0	0	0	0	0
Others	3	1	0	0	4(1.4)

^a^Common Terminology Criteria for Adverse Events (CTCAE) ver.4.0

### Postoperative complications

Postoperative complications in the CRT and the non-CRT groups are presented in Table 6. The presence of retroperitoneal space infection, anastomosis leakage, ileus, urinary dysfunction, and sexual dysfunction were recorded in the clinical findings by a physician who performed imaging analyses (e.g., radiography, CT, fistulography, ultrasonography) and blood examinations. The most common postoperative complication was retroperitoneal space infection, which affected six of 31 (19%) patients in the CRT group; three of these patients underwent radiography-guided drainage. Another patient in the CRT group experienced anastomosis leakage. However, reoperation was unnecessary, as all of the patients who underwent a sphincter-preservation operation (e.g., low anterior resection, ISR, and the Hartman operation) in the CRT group underwent ileostomy, thus preventing major leakage. However, three patients in the non-CRT group received a transverse colostomy. In the CRT group, a conservative therapy was performed in 4 patients with a small bowel obstruction. Urinary and sexual dysfunctions were observed in five of 31 (16%) patients in the CRT group. However, all five patients recovered with medication. None of the patients required permanent self-urethral catheterization. None of the patients in either group died within the first 30 days following surgery.

**Table 6:** Postoperative complications

	CRT(*n* = 31)	non-CRT(*n* = 31)
Retroperitoneal space infection(%)	6(26)	4(27)
Anastomosis leakage(%)	1(4.3)	4(27)
Small intestine obstruction(%)	4(17)	4(27)
Urination and sexual dysfunction(%)	5(22)	3(20)
Mortality(%)	N/A	N/A

N/A not applicable

### Clinicopathological findings

Pathological findings in the CRT and non-CRT groups are presented in Table 7. A pathological complete response (pCR) was observed in two of 31 (6.5%) patients in the CRT group. LLNs metastasis was confirmed in all patients who underwent LLND in both groups.

**Table 7:** The pathological findings in the CRT group and the non-CRT group

	CRT(*n* = 31)	non-CRT(*n* = 31)
Size of tumor(range) [cm]	3.5(0.8-8)	6.0(3-11)
pT(0/1/2/3/4)	2/0/8/20/1	0/0/0/31/0
pN(+/-)	11/20	21/10
Histlogic type(pCR/wel/mod/others)	2/10/17/2	0/27/3/1
TRG (0/1/2/3)	1/13/15/2	N/A
Average number of total resection LN(range)	12(3-33)	12(1-30)
Positive LLNs ratio in LLND case	2(100)	1(100)
Average of distance from anal verge(range)[cm]	4.2(1.5-10)	2(1-5.5)
PM(+/-)	0/31	0/31
DM(+/-)	0/31	0/31
RM(+/-)	0/31	1/30
Adjuvant chemotherapy(%)	13(42)	24(77)

*pT* pathological tumor stages, *pN* pathological node stages, *pCR* pathological complete response, *TRG* tumor regression grade, *LN* lymph node, *LLNs* lateral pelvic lymph nodes, *LLND* lateral lymph node dissection, *PM* proximal margin, *DM* distal margin, *RM* radial margin N/A not applicable

### Short-term outcomes

**Fig. 3:**
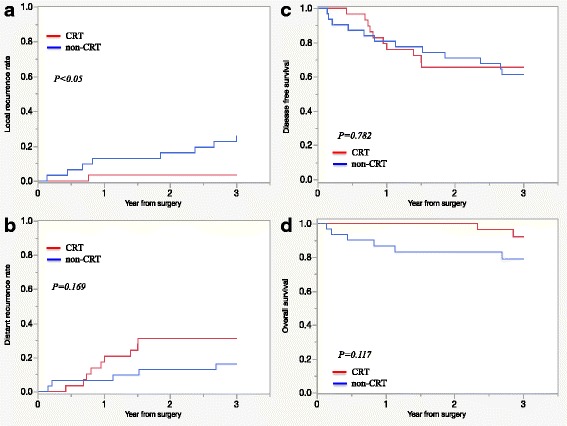
Comparison of clinical outcomes in the two groups. Data were analyzed for 60 patients. **a** 3-year local control rate. **b** 3-year distant control rate. **c** 3-year disease free survival rate. **d** 3-year overall survival rate. Only 29 patients were analyzed in the CRT group, because the follow-up period was too short for the remaining patients

In our study, 29 of 31 patients were analyzed in the CRT group because the follow-up period was too short for the remaining patients. Thirteen of 31 (42%) patients in the CRT group and 24 of 31 (77%) patients in the non-CRT group received adjuvant chemotherapy (Table 7). A significant difference was observed in the estimated 3-year local recurrence rate (LRR) between the CRT and non-CRT groups (*P* < 0.05; Fig. 3a). There were no local recurrences of LLNs. Only one (3.4%) patient in the CRT group had an inner pelvic recurrence. Distant recurrence was detected in nine of 29 (31%) patents in the CRT group and five of 31 (16%) patients in the non-CRT group. No significant difference was observed in the estimated 3-year distant recurrence rate between the two groups (*P* = 0.169; Fig. 3b). The 3-year DFS rates were 66% in the CRT group and 61% in the non-CRT group (*P* = 0.782; Fig. 3c). The 3-year OS rates were 92% in the CRT group and 79% in the non-CRT group (*P* = 0.117; Fig. 3d).

## Discussion

In the present study, we found that CRT with intermittent oral UFT plus LV caused low toxicity (grade 3/4: 9.6%), and achieved a high CRT completion rate (94%) and pCR rate (6.5%), and a low LRR (3.4%).

The Gastrointestinal Tumor Study Group reported the efficacy of adjuvant CRT in 1986 [10, 11]. Intravenous 5-FU plus LV has become the most common CRT regimen for treating rectal cancer. Moreover, chemotherapy with preoperative CRT tends to be a common combination therapy. Several phase III trials have evaluated oxaliplatin in combination with intravenous 5-FU-based CRT, but it was not significantly superior to 5-FU alone [12–15].

As mentioned earlier, the Japanese population that is above the age of 70 is expected to increase. In the current study, more than one-fourth (8/31) of patients were 70 years old or older. The chronological time point that separates elderly from non-elderly patients with cancer is not clearly defined. Bakogeorgos et al. [16] reported that elderly patients with metastatic colorectal cancer equally benefited from treatment compared to their younger counterparts. There is little evidence from clinical studies that elderly patients should be treated with a lower dose of chemotherapy agents.

Unfortunately, UFT treatment in the elderly is limited. However, several trials replaced intravenous 5-FU plus LV with oral UFT plus LV. Roberto et al. [4] summarized the results of clinical trial studies of patients with colorectal cancer who were more than 70 years old and were treated with oral UFT plus LV. These studies confirmed significant safety improvements in the oral UFT plus LV regimen compared to intravenous 5-FU plus LV for treating advanced colorectal cancer. The oral UFT plus LV regimen was better tolerated than the intravenous 5-FU plus LV regimen, but these regimens were equally efficacious in elderly patients and those younger than 65 years old. In addition, preoperative RT (45/50.4 Gy) combined with oral UFT (300–350 mg/m^2^/day) plus LV [17] or oral UFT (400 mg/m^2^/day) alone [18] is as effective as preoperative RT (45-60 Gy) and a continuous venous infusion of 5-FU for treating rectal cancer [19–23]. Phase I [24, 25] and phase II clinical studies [26–29] have evaluated the efficacy and toxicity of oral UFT plus LV with preoperative RT. Grade 3/4 diarrhea was reported in 14–23% of patients treated with preoperative RT plus UFT and LV when using a lower dose of UFT (300 mg/m^2^/day) [15]. In the present study, oral UFT plus LV was administered continuously on days 8–35. We hypothesized that this continuous administration caused severe diarrhea. Compared to Western and European populations, the Japanese population differs in physique and physical strength. Therefore, the optimal dosage and dosage interval must be determined for this population. Several studies examining UFT with CRT for rectal cancer were performed in Caucasian populations. However, the gastrointestinal toxicity of tegafur-based drugs such as UFT and S1 is known to be more tolerable in Asian patients than in Caucasians [30, 31]. In addition, oral UFT plus LV does not cause hand-foot syndrome unlike capecitabine. These are the reasons why oral UFT plus LV has been recommended as adjuvant chemotherapy in the JSCCR guidelines [8] and is commonly selected as one of the chemotherapy agents for rectal cancer patients in Japan; thus we evaluated preoperative CRT with intermittent oral UFT plus LV. Furthermore, few studies have reported the use of preoperative CRT with UFT plus LV for patients with rectal cancer. Our study used an intermittent oral UFT plus LV treatment and patients remained drug-free on days 15–28 (2 weeks), which provided a washout period. Therefore, severe toxicity from oral UFT plus LV could be avoided. As a result, we observed no case of grade 4 toxicity; only three (9.6%) patients had grade 3 diarrhea, and 29 of 31 (94%) patients in the CRT group completed the full course of concurrent CRT and underwent a subsequent operation. We believe that this low level of toxicity and high completion rate associated with our regimen of intermittent oral UFT plus LV led to good outcomes.

In the National Surgical Adjuvant Breast and Bowel Project R03 trial, up to one-third of patients had surgical complications irrespective of whether they received preoperative or postoperative treatment [32]. These findings suggest that preoperative treatment may not increase the surgical complication rate. In our study, surgical complications and outcomes were similar to those reported in the National Surgical Adjuvant Breast and Bowel Project R03 trial. In a Japanese study, Ishihara et al. [33] reported that the addition of oral UFT plus LV did not increase the occurrence of postoperative local septic complications. In our study, the addition of intermittent oral UFT plus LV did not result in an increase in surgical complications associated with preoperative CRT.

In a European multicenter, randomized trial, a statistically significant increase in the DS rate was observed for the long-interval (6–8 weeks) group compared to those undergoing surgery within 2 weeks of completing RT (26% vs. 10%, respectively) [34]. A retrospective study conducted by Moore et al. [35] also exhibited a trend towards a higher pCR and DS rate with increased intervals. On the basis of this evidence, we performed curative operations after a minimum CRT interval of 8 weeks. In our study, the results of the RECIST partial response/complete response and DS rates were 80% and 61%, respectively. Specifically, cT4 and cN2 cases were associated with a high DS rate. According to the pathological findings, a pCR was achieved in 6.5% of patients. The high incidence rate of DS and the pCR rate may be positive factors affecting OS and DFS.

The LRR of rectal cancer is an important factor in determining the patient postoperative outcome. Previous studies have demonstrated that surgical treatment, which consists of TME, is effective for reducing postoperative LRR in patients with rectal cancer [36]. In Japan, the JSCCR guidelines show that TME plus LLND has become the standard treatment for the surgical management of Stage II and Stage III primary rectal carcinomas [8]. Moreover, the postoperative outcomes when performing surgery alone were better than those obtained in Western countries, which is the reason why preoperative CRT has not been introduced aggressively in Japan. However, in some Japanese institutes, CRT with S-1 has been performed as part of a clinical trial. Over 10 years, several phase II studies regarding the safety and efficacy of CRT with S-1 in Japanese advanced rectal cancer patients have been reported [37–39]. However, there are few reports about CRT with oral UFT plus LV for Japanese rectal cancer patients. In the rest of the world, however, since the 2009 National Comprehensive Cancer Network Practice Guidelines were issued, preoperative CRT has become the standard treatment, as RT can reduce the LRR [40, 41] Moreover, RT in combination with TME has also been proven effective for reducing the LRR of patients with rectal cancer [42]. Recently, several studies have demonstrated that preoperative CRT and TME provide a better local control rate than TME and RT alone. However, preoperative CRT did not affect DFS or OS rates in previous studies [43–46]. Wang et al*.* [47] reported that the 3-year OS and DFS rates at a single institution were 92% and 76%, respectively. In our study, the results were comparable to those obtained in the aforementioned study; the 3-year OS and DFS rates were 92% and 78%, respectively. CRT with intermittent oral UFT plus LV caused low toxicity, facilitated a high completion rate of CRT among patients, and provided a better local control rate. These effects may have contributed to the beneficial outcome. In our study, nine patients in the CRT group experienced distant recurrences. Only four of these nine patients received adjuvant chemotherapy. Of the four patients who did not receive adjuvant chemotherapy, three showed DS from clinical stage III to pathological stage II. Distant recurrences in the CRT group may have been a consequence of the reduced adjuvant chemotherapy performed in 13 of 31 (42%) patients in the CRT group. In the future, we will actively consider whether patients should receive adjuvant chemotherapy. Principally, we plan to administer adjuvant chemotherapy to patients with rectal cancer at clinical stage III. Our study did not find a statistically significant difference in the incidence of distant recurrence between the two patient groups. Thus, further progress in the prevention of distant recurrence may be achieved with a more effective chemotherapy regimen.

## Conclusions

Our investigation has limitations, including its retrospective design, small sample size, and short follow-up periods. Our follow-up period was only 3 years, but recurrences of rectal cancer have been observed as late as 5–6 years following completion of the initial treatment. In addition, limited data are available from preoperative CRT studies in Japan on intermittent oral UFT plus LV. In our retrospective study, preoperative CRT with intermittent oral UFT plus LV appears to be a tolerable and effective treatment for Japanese patients with rectal cancer. Future prospective studies are needed to further evaluate the efficacy of preoperative intermittent oral UFT plus LV CRT for treating rectal cancer.

## Abbreviations

5FU: 5-fluorouracil
ASA: American Society of Anesthesiologist
cN: clinical node stages
CRT: Chemoradiotherapy
cT: clinical tumor stages
CT: Computed tomography
DFS: Disease-free survival
DS: Downstaging
ISR: Intersphincteric resection
JSCCR: Japanese Society for Cancer of the Colon and Rectum
LLLD: Lateral lymph node dissection
LLNs: Lateral pelvic lymph nodes
LRR: Local recurrence rate
LV: leucovorin
MRI: Magnetic resonance imaging
OS: Overall survival
pCR: pathological complete response
RESIST: Response Evaluation Criteria in Solid Tumors
RT: Radiotherapy
TME: Total mesorectal excision
UFT: Tegafur-uracil
UICC: Union for International Cancer Control
