# Erratum to: Efficacy and short-term outcomes of preoperative chemoradiotherapy with intermittent oral tegafur-uracil plus leucovorin in Japanese rectal cancer patients: a single center experience retrospective analysis

**DOI:** 10.1186/s12957-017-1224-2

**Published:** 2017-09-15

**Authors:** Ryosuke Nakagawa, Yuji Inoue, Takeshi Ohki, Yuka Kaneko, Fumi Maeda, Masakazu Yamamoto

**Affiliations:** Department of Surgery, Institute of Gastroenterology, Tokyo Women’s Medical University, 8-1 Kawada-cho, Shinjuku-ku, Tokyo, 162-8111 Japan

## Erratum

Upon Publication of the original manuscript [1] several discrepancies were highlighted in both the Abstract and Tables 5 and 6. The errors in tables 5 and 6 have since been acknowledged and corrected in this erratum. This manuscript was also updated to amend the abstract. It was highlighted that errors were present in both tables 5 and 6. The values given in these tables were incorrect and were misleading to the reader. These errors change neither the outcome of the experiments nor the conclusions of the article.

The corrected tables are shown below:

**Table 5:** Toxicity Grade^a^ patients with CRT (*n* = 31)

	Grade 1	Grade 2	Grade 3	Grade 4	total (%)
Diarrhea	6	9	3	0	18 (58)
Radiation dermatitis	4	2	0	0	6 (19)
Nausea	1	0	0	0	1 (3.2)
Leucopenia	0	0	0	0	0
Others	3	1	0	0	4 (13)

^a^Common Terminology Criteria for Adverse Events (CTCAE) _ver.4.0_

**Table 6:** Postoperative complications

	CRT (*n* = 31)	non-CRT (*n* = 31)
Retroperitoneal space infection (%)	6 (19)	4 (13)
Anastomosis leakage (%)	1 (3.2)	4 (13)
Small intestine obstruction (%)	4 (13)	4 (13)
Urination and sexual dysfunction (%)	5 (16)	3 (9.6)
Mortality (%)	N/A	N/A

N/A not applicable
